# Shared decision-making in urinary reconstruction among radical cystectomy patients: a thematic analysis of patient and provider perspectives in China

**DOI:** 10.1186/s12885-025-15052-5

**Published:** 2025-10-15

**Authors:** Wang Li, Yiqin Shi, Liping Pu, Junyong Zhang, Mingyan He, Jianxia Chen, Xiaoqin Huang

**Affiliations:** Department of Urology, The Second Affiliated Hospital of Chongqing Medical University, Chongqing, 400010 China

**Keywords:** Qualitative research, Shared decision-making, Urinary reconstruction, Radical cystectomy, Doctor-patient relationship, Chinese healthcare system

## Abstract

**Background:**

Shared decision-making (SDM) improves outcomes in urinary reconstruction after radical cystectomy, but its implementation in China remains understudied. This qualitative study explores SDM barriers and facilitators from patient and provider perspectives.

**Methods:**

This study employed purposive sampling to recruit healthcare professionals and patients undergoing radical cystectomy from a single tertiary hospital. A qualitative approach involving semi-structured interviews was used to capture participants’ perspectives. Thematic analysis was applied to identify key themes and subthemes.

**Results:**

A total of 11 healthcare professionals and 15 patients with bladder cancer participated. Thematic analysis identified 4 main themes and 15 subthemes. (1) Decision autonomy and experience: encompassing patients’ participation levels, confidence, and anxiety, and providers’ attention to patient trust and preferences. (2) Information acquisition and understanding: including patients’ information sources and comprehension abilities; and methods used by healthcare professionals to communicate medical information. (3) Professional expertise and occupational burden: exploring patients’ perceptions of doctors’ authority and skills; with role strain and emotional challenges faced by providers. (4) Social security and education: addressing gaps in health literacy; protection of patient rights; and inadequacies in SDM-related education.

**Conclusions:**

SDM in China is limited by systemic challenges, including health literacy deficits, clinician workload, and policy gaps. Targeted interventions such as patient education, communication training, and policy reforms are needed to enhance collaborative decision-making.

## Background

Bladder cancer is a prevalent urological malignancy, which poses significant threats to health and survival in China [1, 2]. Among the diagnosed cases of bladder cancer, muscle-invasive bladder cancer accounts for 25–30% [3, 4] and is prone to progression and metastasis. Radical cystectomy with urinary diversion is a common treatment for muscle-invasive and select high-risk non-muscle-invasive bladder cancer [4]. Patients are presented with various options for urinary diversion and reconstruction before surgery, requiring consideration of risk-benefit ratios, outcomes, survival time, survival rates, and quality of life [5–9]. Occasionally, it is difficult to find the most suitable treatment approach, which makes the clinical decision-making process increasingly complex. Balancing life expectancy and quality of life requires patients to participate in clinical treatment decisions and choose therapies that best align with their personal values [10, 11]. One of the duties of urological specialists and nurses is to assist patients in making these decisions [11, 12]. How to fully leverage the bridging roles of chronic disease specialists and nurses to facilitate effective therapeutic communication between patients and doctors remains to be explored through research.

Shared decision-making (SDM) refers to a process in which healthcare professionals and patients jointly access medical and nursing information [13, 14]. This collaborative approach allows mutual decision-making between patients and healthcare professionals [15–17]. Research has demonstrated that involving patients in the entire disease management process can assist healthcare professionals in obtaining patient-specific treatment information, enhance communication between doctors and patients, improve disease management, and reduce healthcare costs [13, 18, 19]. Recent Chinese studies highlight growing SDM adoption in tertiary hospitals but note persistent barriers, including physician time constraints and cultural preferences for clinician guidance in surgical decisions [18, 20]—particularly relevant for urinary reconstruction’s complex options. Within the technically complex arena of urinary reconstruction, Confucian values that prioritize hierarchical relationships profoundly inform Chinese clinical interactions, often serving to amplify inherent power imbalances.

Qualitative research can provide an in-depth understanding of clinical decision-making and explore various types of information related to event occurrence [21, 22]. While SDM has been extensively studied in Western contexts, qualitative research specifically examining urinary reconstruction decisions remains scarce in China. As one of the first qualitative studies in this area, it is conducted with the ultimate goal of improving patient participation in treatment decision-making for bladder cancer.

## Materials and methods

### Study design

This study adopted a qualitative research design to explore the multifaceted factors influencing decision-making regarding urinary reconstruction. Guided by a constructivist paradigm, which posits that individuals construct their own understandings and experiences of reality through social interaction, It was designed to capture participants’ experiences and perspectives, focusing on their preferences, challenges, and barriers encountered during SDM. To achieve this, we conducted semi-structured interviews that encouraged the participants to share their personal stories and emotions. The collected data were analyzed using an inductive thematic analysis approach, this approach was selected for its demonstrated effectiveness in: (1) identifying patterns across complex qualitative datasets, (2) generating clinically actionable insights from patient-provider interactions, and (3) accommodating diverse perspectives inherent in SDM research [21, 22]. Throughout the study, we adhered to the guidelines outlined in the consolidated criteria for reporting qualitative research (COREQ) checklist to ensure standardization and methodological rigor [23]. All participants provided informed consent before participation. This study was approved by the Ethics Committee of the Second Affiliated Hospital of Chongqing Medical University (Approval No. 2021 − 282) and conducted in compliance with the ethical principles outlined in the Declaration of Helsinki.

### Participants

To obtain a comprehensive perspective on SDM, we recruited urological healthcare professionals and patients with bladder cancer from the affiliated hospital of a medical university in Chongqing between January 2023 and July 2023. Participants were selected using purposive sampling based on the following inclusion criteria: (1) aged ≥ 18 years at the time of the interview; (2) provision of oral informed consent; (3) cognitive ability to communicate effectively and participate in qualitative interviews; (4) for patients, a clinical diagnosis of muscle-invasive bladder cancer (MIBC) or other high-risk bladder malignancies eligible for radical cystectomy; and (5) for healthcare professionals, a minimum of 1 year of relevant work experience. Patients in postoperative recovery and those with unstable health conditions, neurological or psychiatric disorders, or significant communication impairments were excluded. Potential selection biases (urban skewness inherent to single-center designs, distress-related non-participation, provider self-selection) were mitigated through maximum variation sampling across demographic and professional characteristics. Data saturation was determined to be achieved when analysis of three consecutive interviews yielded no new themes or subthemes, indicating thematic redundancy. This criterion confirmed the adequacy of the sample size (26 participants), consistent with qualitative research norms [22].

### Data collection

Data were collected through semi-structured in-depth interviews designed to gather detailed insights into the participants’ perceptions of decision-making in urinary reconstruction. The principal interviewer (LW), trained in qualitative methodologies, conducted all interviews. To ensure consistency and depth, the semi-structured interview guide was developed through a systematic process involving: a comprehensive literature review to identify core SDM domains in urologic oncology, iterative consultations with urology specialists and patients to refine question relevance, and pilot testing with patients and providers to optimize clarity and flow. The interview guide comprised open-ended questions designed to explore participants’ experiences, perspectives, and challenges related to SDM. For instance, patients were asked, “How do you collaborate with your doctor to make surgical decisions?” and “What difficulties did you encounter during the decision-making process?” to elicit detailed accounts of their understanding of bladder cancer, treatment options, and influencing factors such as risks, benefits, and financial considerations, as well as their emotional experiences throughout the process. For healthcare providers, sample questions included, “What are your perspectives on patients’ participation in shared surgical decision-making?” and “What difficulties did you encounter during the decision-making process?“, aiming to capture their approaches to facilitating SDM, managing time constraints, and incorporating patient preferences. The guide allowed flexibility for follow-up questions and emerging themes, ensuring comprehensive yet adaptable interviews. A complete version of the interview guide is available in Table 1. The interviews were conducted in a quiet, private environment to ensure confidentiality and minimize distractions. Throughout the interviews, informal member-checking was practiced. Interviewers periodically summarized participants’ statements to verify interpretive accuracy in real-time. Each interview lasted 30–60 min and was audio-recorded with the participants’ consent. Recruitment and data collection were continued iteratively until data saturation was reached.

**Table 1:** Interview outline

Bladder Cancer Patients	Healthcare Providers
① What are your perspectives on urinary reconstruction surgery?	① What are your perspectives on patients’ participation in shared surgical decision-making?
② How do you collaborate with your doctor to make surgical decisions?	② How do you assist patients in making decisions?
③ What difficulties did you encounter during the decision-making process? And how did you overcome these challenges?	③ What difficulties did you encounter during the decision-making process? And how did you overcome these challenges?
④ How did you feel after making the decision?	④ What kind of feedback did you receive after the decision was made༟
⑤ What factors can assist you in developing better solutions?	⑤ What factors can contribute to patients making better decisions?
⑥ How do you view shared decision-making?	⑥ How do you view shared decision-making?
⑦ How do you access and utilize patient decision aids?	⑦ How do you access and utilize patient decision aids?
⑧ Is there anything else you would like to share with me or tell me?	⑧ Is there anything else you would like to share with me or tell me?

### Data analysis

The recorded interviews were transcribed verbatim, and two researchers (LW and SYQ) independently reviewed and crosschecked the transcripts to ensure accuracy. Two researchers independently conducted initial open coding using NVivo software, a standard tool for ensuring transparency in qualitative analysis, to identify key decision-making segments, with regular meetings to compare and reconcile coding frameworks until achieving consensus. These codes were organized into higher-order categories to develop preliminary themes. Themes were refined through constant comparison and axial coding to establish relationships between categories [24]. To ensure intercoder agreement, two researchers (LW and PLP) independently coded a transcript subset and resolved discrepancies through reconciliation meetings. Final themes were validated through team discussions to ensure consistency and validity. Member-checking was employed to validate the findings, allowing participants to review and confirm the interpretations of their responses. Triangulation was used to compare the perspectives of patients and healthcare professionals, ensuring a comprehensive and balanced analysis. Throughout the process, the existing literature was referenced to contextualize the findings and identify areas of alignment or divergence. To enhance reliability and rigor, the research team collaborated through all stages of the analysis to minimize individual bias. The results were presented through detailed thematic descriptions and visual representations, highlighting the complex interplay of factors influencing decision-making in urinary reconstruction.

## Results

**Fig. 1:**
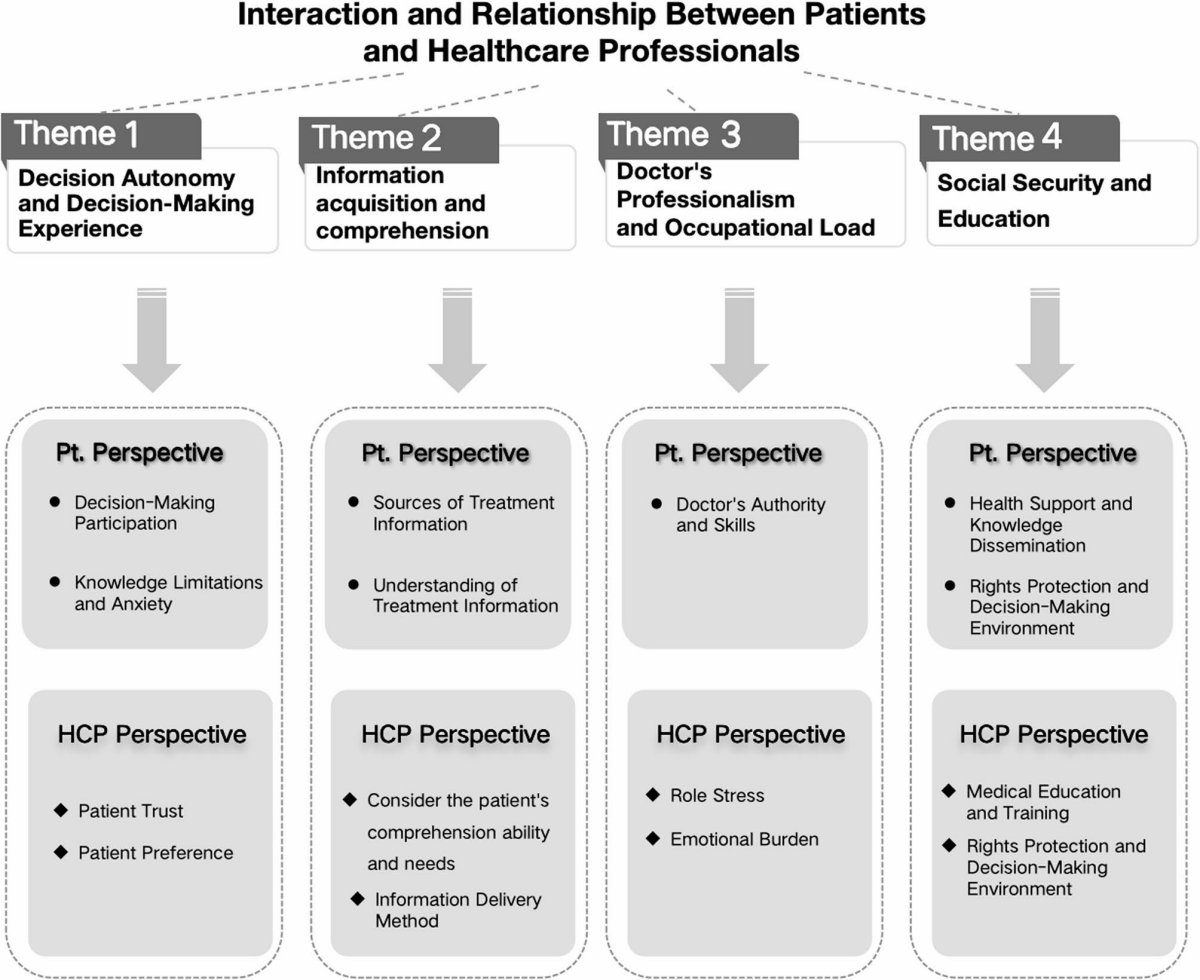
Patient and health care provider perspectives on vascular access–related decision-making Note. *Pt. Perspective* Patient Perspective, *HCP Perspective* Healthcare Professional Perspective

This study involved 26 participants, including 15 patients who had undergone radical cystectomy with urinary reconstruction and 11 healthcare providers (8 physicians and 3 nurses). The patients ranged in age from 50 to 75 years, with 13 males and 2 females (Table 2). Healthcare professionals had 5–31 years of clinical experience (Table 3). Using in-depth interviews and thematic analysis, the study identified four key themes: (1) autonomy and decision-making experience; (2) access to and comprehension of information; (3) physician expertise and workload; and (4) the influence of social security and education. Each theme comprises multiple subthemes. To better illustrate these dynamics, a schematic diagram (Fig. 1) was developed that visually mapped the relationships between the four themes and subthemes. This framework highlights the complex and interactive processes involved in decision-making for urinary reconstruction and provided insights into the factors that shape patient-provider collaboration.

**Table 2:** Characteristics of patients (*n* = 15)

Demographic characteristic	*n* = 15
Gender	
Male	13
Female	2
Age (years)	
Below 50	1
50–64	9
65 or above	5
Education Level	
Primary school	1
Middle school	2
High school	5
Vocational training	2
Bachelor’s degree	4
Postgraduate degree	1
Employment Status	
Employed	5
Self-employed	1
Unemployed	1
Retired	7
Homemaker	1
Marital Status	
Married	12
Divorced	1
Widowed	1
Single	1
Living Arrangement	
With spouse	10
With spouse and child	2
With adult children	1
Alone	2
Time Since Radical Cystectomy(months)	
3–12	13
13–24	2
Type of Urinary Reconstruction	
Ileal conduit	11
Neobladder	4
Ureterostomy	1

**Table 3:** Characteristics of health care provider participants (*n* = 11)

Characteristic	*n* = 11
Role	
Doctor	8
Nurse	3
Gender	
Male	8
Female	3
Age (years)	
Below 40	6
41–50	3
51 or above	2
Clinical Experience (years)	
5 or less	1
6–10	2
11–20	6
21–30	1
Above 30	1
Time Dedicated to Clinical Work (%)	
Above 50	10
25–50	0
Less than 25	1

### Decision autonomy and decision-making experience

In the SDM process for urinary reconstruction, notable differences exist between patients and healthcare providers regarding their perceptions of decision-making autonomy and experience. Patients reported varying degrees of pressure and anxiety, while providers emphasized patient trust and preferences. Two subthemes were identified: (1) patient decision participation, knowledge limitations, and anxiety; and (2) patient trust and preferences from the provider perspective.

#### Patient perspective: decision participation, confidence, and anxiety

Patients frequently described passive decision-making roles due to:

##### Cultural norms of respect for authority

Patients often deferred to physicians due to cultural norms of respect for authority.

*“ I had no choice but to trust them—it’s their expertise*,* after all.” (P3)*.

##### Emotional impacts on decision-making

Anxiety frequently compounded these knowledge gaps:

*“ I’m scared I won’t be able to return to my previous state.” (P15)*.

*“I kept going over the options*,* but no matter what I thought*,* every decision felt stressful.”(P8)*.

#### Healthcare provider perspective: patient trust and preferences

##### Patient trust

Healthcare providers emphasized that trust is fundamental to effective SDM.

*“Building trust isn’t instant—it requires ongoing and sincere communication.” (D3)*.

Providers observed that trust could simplify the decision-making process because patients who trust their care team are more likely to accept recommendations without hesitation.

*“When patients trust us completely*,* they’re more open to suggestions*,* and the whole process becomes smoother.” (N1)*.

However, some providers have noted that excessive reliance on trust may undermine patient autonomy, potentially leading to postsurgical regret or doubt.

##### Patient preferences

While offering medically appropriate recommendations, providers recognized the importance of accounting for individual factors, such as lifestyle, cultural background, and psychological state.

*“Understanding their preferences helps us design more personalized treatment plans.” (D2)*.

Providers occasionally face challenges in aligning patient preferences with the optimal clinical recommendations.

*“Some patients prefer less invasive but less effective options*,* and we need to spend extra time explaining the trade-offs.” (D5)*.

### Information access and comprehension

Information transparency, accessibility, and clarity significantly affect patients’ decision-making abilities and engagement levels. This study identified two subthemes: (1) sources of treatment information and comprehension of treatment options from the patient’s perspective, and (2) healthcare providers’ considerations of patient understanding and needs, along with their chosen communication strategies.

#### Patient perspective: sources of treatment information and comprehension

##### Sources of treatment information

Patients accessed information from various sources including explanations provided by healthcare providers, online resources, and shared experiences with other patients.

*“Some terms were too technical to grasp fully.” (P8)*.

*“Online resources provide plenty of information*,* but some of it is contradictory and hard to verify.” (P14)*.

*“Hearing the experiences of others gave me a better understanding of life after surgery.” (P9)*.

However, others found such exchanges anxiety-inducing.

*“Listening to negative experiences made me second-guess my decisions.” (P15)*.

##### Comprehension of treatment information

Many patients highlighted patient preferences for visual aids.

*“I prefer diagrammatic representations rather than written documentation” (P9)*.

Patients with higher education levels felt more confident in engaging with medical information, whereas others struggled.

*“I could discuss the options with the doctor because I had previous exposure to similar concepts.” (P7)*.

A patient with a lower level of education,

*“I couldn’t follow the doctor’s explanation at all.” (P3)*.

Anxiety often hindered patients’ ability to comprehend information.

*“Anxiety made it hard to absorb the information.” (P10)*.

#### Healthcare provider perspective: considering patient understanding, needs, and communication methods

##### Considering patient understanding and needs

Healthcare providers acknowledged that patients’ ability to process information varies greatly and emphasized the need to tailor communication to individual needs.

*“We simplify explanations and use visuals to convey risks and options more effectively.” (D6)*.

Providers recognized that patients often sought information about their lives after surgery, including their ability to work or travel. As one nurse stated,

*“Patients don’t just ask about success rates; they want to know how surgery will impact their everyday lives.”(N3)*.

##### Communication methods

Providers employed multiple communication strategies, including face-to-face discussions, written materials.

Real-time conversations allowed providers to directly address patients’ concerns.

*“Seeing patients’ reactions helps ensure they truly understand the information.” (D1)*.

However, the time constraints were a recurring challenge.

*“We have limited time with each patient*,* so we must prioritize the most critical points.”(D3)*.

Written and multimedia resources were considered effective supplements, depending on patient preferences.

*“Videos and diagrams make complex procedures easier to understand*,* but older patients often prefer verbal explanations.” (N1)*.

Providers emphasized the importance of two-way communication.

*“When patients ask questions*,* it helps us identify their key concerns and provide more targeted explanations.” (D1)*.

### Physicians’ professional competence and occupational burden

Patients emphasized physicians’ authority and skill, while providers reported role pressures and emotional burden.

#### Patient perspective: physicians’ authority and skills

Patients describing how providers’ clinical expertise and interpersonal approach influenced decisions:

*“Hearing that the doctor had performed many similar surgeries reassured me.” (P7)*.

*“The doctor seemed experienced*,* but their explanation felt rushed*,* like I was just another case to handle.” (P10)*.

This trust was often rooted in patients’ self-perceived knowledge limitations:

*“I don’t know much about the surgery*,* so I have to trust the doctor.”(P3)*.

#### Healthcare provider perspective: role pressures and emotional burden

##### Role pressures

Providers highlighted the challenge of dedicating sufficient time to discuss treatment options with every patient while adhering to their busy schedules.

*“We see so many patients each day. Although I’d like to provide detailed explanations*,* it’s not always feasible.” (D3)*.

Thematic analysis revealed that team-based strategies were pivotal in facilitating SDM implementation.

*“Nurses took primary responsibility for preoperative education.”(N1)*.

Providers described the strain of addressing patients’ high expectations regarding surgical outcomes.

*“Patients frequently expect perfect results*,* but every surgery involves risks. We constantly work to align their expectations with reality.” (N2)*.

##### Emotional burden

Providers frequently empathize with patients’ fears and anxieties, which, while fostering trust, lead to emotional exhaustion. Patient frustration or anxiety was sometimes directed at healthcare providers, exacerbating their emotional burden.

*“Constantly dealing with worries is draining.” (D4)*.

*“Patients sometimes blame us when they don’t fully understand the situation. It can feel overwhelming and frustrating.” (N2)*.

Some providers used team discussions or counseling as coping strategies to manage emotional challenges.

*“Discussing complex cases with the team helps me feel supported.” (D4)*.

### Social support and education

We identified subthemes including patients’ perspectives on health knowledge dissemination, rights advocacy, and healthcare providers’ perspectives on SDM education, training, and policy-driven decision-making environments.

#### Patient perspective: health knowledge dissemination and advocacy for rights in decision-making environments

##### Health knowledge dissemination

The study revealed that the quality and accessibility of health education influenced patients’ decision-making capacity and confidence.

*“Before deciding on the surgery*,* I felt completely lost because no one explained what options I had or the consequences of those options.” (P13)*.

Familial scaffolding emerged as a critical enabler of effective SDM, particularly for patients navigating complex urinary reconstruction options.

*“My family helped me look up information.” (P15)*.

##### Advocacy for rights and decision-making environments

Many patients lacked a clear understanding of their rights in medical decision-making, which resulted in a passive role in the information exchange process.

*“I didn’t know if I had the right to ask more questions or choose a different treatment.” (P9)*.

*“Some surgeries require additional costs*,* but I wasn’t sure if they would be reimbursed*,* so I had to choose the most affordable option.” (P3)*.

Some patients called for more transparent decision-making support systems.

*“If there were a dedicated consultation service to help us understand the surgical options and costs*,* that would be a great help.” (P6)*.

#### Healthcare provider perspective: SDM education, training, and advocacy for rights in decision-making environments

##### SDM education and training

Healthcare providers identified a lack of SDM knowledge and formal training as major barriers to its clinical implementation. To address this gap in medical education, they recommended reinforced training through interdisciplinary collaboration, case-based learning, and simulated scenarios.

*“We understand that patients need to be involved in decision-making*,* but sometimes we don’t know how to make it happen effectively.” (D7)*.

*“In school*,* we focused mostly on clinical procedures. Communication and decision-making training were very limited.” (N3)*.

*“If we had more courses that simulate patient communication*,* our skills would definitely improve.” (D3)*.

##### Advocacy for rights and decision-making environments

Healthcare providers pointed out that some medical policies and procedures limit the depth of patient involvement in decision-making. Additionally, providers emphasized that the current allocation of medical resources often hinders meaningful patient interactions.

*“Certain treatment plans require approvals from multiple departments*,* but these processes often overlook patient needs.” ((D6)*.

*“We have so many patients to care for that it’s impossible to spend enough time listening to each one’s opinion.” (N1)*.

Providers suggested simplifying procedures, optimizing resource allocation, and introducing patient rights advocacy mechanisms to improve decision-making environments.

*“We could establish patient rights advocates to ensure that patients have opportunities to express their views at every stage of the decision-making process.” (D8)*.

## Discussion

### Key findings compared to existing literature

This qualitative study examined the multitude of factors influencing SDM in urinary reconstruction surgery through integrated analysis of patient and healthcare provider perspectives. Four major themes and multiple subthemes were identified, shedding light on the distinctive challenges and opportunities of SDM in China’s healthcare system. These findings contrast with national and international literature [13, 19, 20].

#### Autonomy and decision-making experience

Findings from the patient perspective: This study highlighted significant differences between Chinese patients and those in Western countries in terms of participation and autonomy in decision-making. Patients often experienced decision-making anxiety, and in some cases, regretted decisions [14, 25]. Unlike studies that emphasize active patient participation [26], many Chinese patients relied on the advice of their doctors because of limited medical knowledge, insufficient understanding of surgical options, and inadequate time to process information, leading to a passive decision-making role [18, 27–29]. This situation is not unique to China, and is also observed in other countries with a high degree of doctor-patient authority inequality [30, 31], where patients depend on professional judgment, even when multiple options are available. Compared with Western models that encourage active patient involvement in decision-making [32, 33], many patients still felt that their decision-making capacity was limited and often made decisions under the guidance of their doctors. This phenomenon of doctor-patient authority dependence is common in cultures where doctor-patient relationships emphasize authority, and not only in China [34, 35].

Findings from the healthcare provider perspective: Healthcare providers play a significant role in supporting patient autonomy [33, 36]. This study found that the providers’ understanding of patients’ preferences and values directly affected patients’ decision-making experience. In Chinese healthcare, owing to time and resource constraints, doctors often provided brief explanations of the risks and benefits of surgery while neglecting the patients’ emotional needs and personal values, thus limiting the depth of SDM [16, 30, 37].

#### Information access and comprehension

Findings from the patient perspective: Information access and understanding are central to SDM. Patients’ ability to access and comprehend information significantly affects SDM quality [38]. This study revealed that many patients relied heavily on physicians’ explanations to understand surgical options, with limited initiative to seek information from other sources. Compared to patients in developed countries, who commonly gather medical information from multiple channels such as the internet and patient support groups [39], Chinese patients had limited sources of health information [27], because of their lower health literacy and limited information access [40], particularly in economically disadvantaged regions where understanding complex medical information remains a challenge [30, 31].

Findings from the healthcare provider perspective: This study emphasized the critical role of healthcare providers in information delivery [13]. Doctors often adjusted their communication strategies based on the patient’s cultural background, educational level, and psychological state, opting for simplified presentations of key information. While this approach facilitated quicker comprehension within limited consultation times, it sacrificed the complexity of the information, resulting in patients having an incomplete understanding of the surgical risks and expected outcomes [17]. This, in turn, impacted decision-making quality. This highlights the urgent need for improved health education, greater transparency, and accessibility of information, particularly regarding complex treatment decisions [40].

Overall, this study revealed the dual challenges of information access and understanding including patients’ health literacy and comprehension abilities and the communication strategies employed by healthcare providers in high-pressure environments. Future efforts should focus on improving health education, enhancing accessibility of information, and training healthcare providers in effective communication to better support patients and improve SDM implementation.

#### Physician expertise and professional workload

Findings from the patient perspective: Physicians’ expertise and experience play decisive roles in the decision-making process [41]. This study found that Chinese patients placed a high degree of trust in their doctors’ professional competencies, especially when facing complex treatment decisions, often delegating decision-making authority to doctors. This contrasted with the tendency of patients in high-income Western countries to be more actively involved in decision-making [12]. Chinese patients tended to focus on the doctors’ experience and skills rather than an active discussion of the surgical plan [42, 43].

Findings from the healthcare provider perspective: Consistent with international studies, this study confirmed that physicians’ expertise and authority are crucial to the decision-making process [44–46]. However, it also revealed the pressure and emotional burden faced by doctors owing to heavy workloads. Compared with their international counterparts, Chinese doctors saw a significantly higher number of patients, which limited the time available for in-depth discussions. This finding aligns with domestic research highlighting the role of professional workload in shaping doctor-patient dynamics and SDM quality [47, 48]. Furthermore, Chinese doctors often bore a heightened sense of responsibility in the decision-making process, likely because of patients’ reliance on medical authority [49, 50]. Addressing these dynamics requires efforts to improve providers’ working conditions, enhance access to emotional support resources, and strengthen communication skills through targeted training. These measures can help mitigate existing challenges and improve the overall quality and effectiveness of SDM.

#### Social support and education

Findings from the patient perspective: Social support and health education are key factors influencing patient involvement in SDM [14, 39, 51], yet our findings reveal significant gaps in China’s implementation. Many patients lacked understanding of complex surgeries and insurance policies, reflecting systemic inadequacies in knowledge dissemination [52] and diverging from international evidence linking policy transparency to decision confidence [35, 38]. These results align with but extend classic SDM frameworks [26] by identifying structural policy barriers (e.g., insurance limitations) and cultural norms (e.g., deference to physician authority) as critical themes within China’s context. The observed information asymmetry and limited postoperative support underscore the need for context-sensitive SDM strategies that address both structural health system constraints and patient education gaps.

Findings from the healthcare provider perspective: In contrast to the well-established SDM education systems in other countries, healthcare providers in China are still in the early stages of training and education, which hinders the deeper integration of SDM into clinical practice [53, 54]. This study found that, while healthcare providers tried to convey information in clinical practice, they often lacked systematic training in SDM, leading to incomplete patient understanding of treatment options and, at times, insufficient informed decision-making. This contrasts sharply with mature SDM training frameworks in developed countries [51, 53, 55].

Therefore, improving health literacy and decision-making abilities among patients is essential, particularly regarding financial burdens and treatment options. Future practice should enhance patient education through easily understandable materials and multi-channel information dissemination to improve accessibility and transparency. Concurrently, SDM training for providers must be strengthened, especially in communicating complex information, assessing psychological needs, and offering emotional support. System-level improvements—such as insurance reforms and pilot SDM programs tailored to China’s healthcare structure—should be grounded in realistic feasibility assessments, accounting for current resource constraints and policy frameworks. Enhancing social support and education can effectively promote more active and inclusive SDM processes.

### Strengths and limitations

This study offers several strengths including its multi-perspective approach integrating both patient and healthcare provider views to comprehensively explore SDM factors in urinary reconstruction surgery, its grounding in China’s unique healthcare and sociocultural context that provides locally relevant insights, and its structured thematic analysis that developed a theoretical framework for future research. However, these findings should be interpreted in light of certain limitations: the single-institution sampling may not capture regional variations across China, potential information bias from subjective participant accounts could affect interaction dynamics representation, and the inherent lack of generalizability in qualitative methods necessitates future quantitative validation studies to assess broader applicability. While we employed rigorous analytical methods, the study’s findings may have been influenced by the researchers’ clinical backgrounds in urology, which could have shaped both the data collection process and the interpretation of participants’ perspectives on medical decision-making. Furthermore, reflexivity was considered regarding the research team’s clinical background in urology and nursing, which may have influenced data interpretation and thematic coding. To mitigate potential bias, regular team discussions were used to critically examine presuppositions. Regarding transferability, findings related to distinct sociocultural norms—such as pronounced deference to physician authority—and unique insurance-based constraints are particularly reflective of China’s healthcare context and may have limited applicability in settings with different medical systems and cultural values. However, themes surrounding the universal need for clearer information, empathetic communication, and structured emotional support are likely to resonate across diverse environments, especially those undergoing comparable healthcare reforms aimed at enhancing patient-centered care.

### Implications for practice

Based on the findings of this study and the unique characteristics of China’s healthcare system, the following practical recommendations are proposed:

#### Strengthen patient health education

Health education is critical in enhancing patients’ decision-making capabilities. Governments and healthcare institutions should intensify their efforts to promote health literacy in urological surgeries. This includes developing accessible educational materials and disseminating surgical information through diverse channels such as hospital seminars, online courses, and community activities. These initiatives will enable patients to participate more effectively in the decision-making process.

#### Optimize communication training for healthcare providers

Healthcare providers play an indispensable role in SDM. Targeted training modules should be incorporated into medical education and continuing professional development programs to improve providers’ communication skills, emotional support capabilities, and SDM techniques.

#### Reforming insurance policies

Governments should focus on improving the medical insurance system to provide patients with clear and transparent policy support during treatment decisions.

#### Reducing provider workload

Healthcare institutions should adopt strategies to reduce physicians’ occupational stress, such as increasing staffing levels, optimizing workflows, and redistributing resources.

#### Adopting digital health tools

Recommended digital health measures include integrating structured decision aids and implementing telemedicine platforms. These tools should be incorporated into routine clinical pathways to support information delivery and remote consultation.

#### Expanding SDM awareness

As a novel concept in China, SDM requires further dissemination and policy support. Pilot SDM projects can be established in hospitals to integrate SDM into clinical workflows. Tailoring implementation strategies to align with local patient needs and resource conditions will help to identify pathways suited to China’s healthcare environments.

Building upon the logic model (Fig. 1), we propose tiered implementation strategies: in tertiary hospitals, nurse-led SDM pathways and digital decision aids can address time constraints, while rural settings may benefit from family-mediated education and telemedicine to overcome health literacy and specialist access barriers.

By analyzing the key factors influencing SDM in urinary reconstruction surgery, this study provides theoretical insights and practical recommendations for improving medical practice in China. Future efforts must involve a coordinated approach to advance health education, enhance social security, and optimize medical resources to create a more harmonious, transparent, and supportive decision-making environment for patients and healthcare providers.

## Conclusion

This study highlights cultural, informational, and systemic barriers to SDM in urinary reconstruction in China. Targeted interventions—improving health literacy, clinician communication training, and policy reforms—are needed to foster collaborative decision-making and improve outcomes.

## Data Availability

The data that support the findings of this study are available from the corresponding author upon reasonable request.

## Abbreviations

COREQ: Consolidated criteria for reporting qualitative research
MIBC: Muscle-invasive bladder cancer
SDM: Shared decision-making
